# Evaluating Functional Connectivity Alterations in Autism Spectrum Disorder Using Network-Based Statistics

**DOI:** 10.3390/diagnostics8030051

**Published:** 2018-08-07

**Authors:** Aitana Pascual-Belda, Antonio Díaz-Parra, David Moratal

**Affiliations:** Centro de Biomateriales e Ingeniería Tisular, Universitat Politècnica de València, 46022 Valencia, Spain; aitanapasbel@gmail.com (A.P.-B.); antodipar@gmail.com (A.D.-P.)

**Keywords:** Autism Spectrum Disorder, functional MRI, resting-state, brain connectivity, complex networks, Network-Based Statistic, overconnectivity, underconnectivity

## Abstract

The study of resting-state functional brain networks is a powerful tool to understand the neurological bases of a variety of disorders such as Autism Spectrum Disorder (ASD). In this work, we have studied the differences in functional brain connectivity between a group of 74 ASD subjects and a group of 82 typical-development (TD) subjects using functional magnetic resonance imaging (fMRI). We have used a network approach whereby the brain is divided into discrete regions or nodes that interact with each other through connections or edges. Functional brain networks were estimated using the Pearson’s correlation coefficient and compared by means of the Network-Based Statistic (NBS) method. The obtained results reveal a combination of both overconnectivity and underconnectivity, with the presence of networks in which the connectivity levels differ significantly between ASD and TD groups. The alterations mainly affect the temporal and frontal lobe, as well as the limbic system, especially those regions related with social interaction and emotion management functions. These results are concordant with the clinical profile of the disorder and can contribute to the elucidation of its neurological basis, encouraging the development of new clinical approaches.

## 1. Introduction

The use of functional magnetic resonance imaging (fMRI) to evaluate resting-state functional brain networks has largely contributed to broaden the study possibilities of neurological disorders, such as Autism Spectrum Disorder (ASD), Alzheimer Disease and schizophrenia, among others [1]. More concretely, measuring the intrinsic fluctuations in Blood Oxygen Level Dependent (BOLD) signals has made it possible to determine which brain regions are synchronized and, using inter-group comparisons, to clarify if there exist significant differences in the way these regions interact under different neurological conditions [2]. The aim of this work is to evaluate these interactions among different brain regions in subjects affected by the ASD, in order to determine if their brain regions synchronization patterns show a remarkable alteration when comparing them to neurologically typical (or neurotypical) subjects. Previous studies have not been able to reach a consensus regarding to the way in which ASD subjects’ brain patterns are altered: Whereas some studies determine that brain connectivity patterns in subjects affected by the ASD show a brain-wide underconnectivity, there are other studies that claim that ASD subjects show a generalized overconnectivity, compared with neurotypical subjects. Moreover, other studies support the hypothesis of the combination of both under- and overconnectivity. Hull et al. [3] reviewed ASD-connectivity-related literature, and its review reaffirmed the lack of consensus in the scientific community towards this point.

In this study, the approach that has been used is that of network science [4]. A network is a representation of a real-world complex system, and is defined through a set of nodes or regions, connected among them through a set of links or edges [5]. Macroscopically, these nodes or regions can be defined according to anatomical or functional criteria, whereas the connectivity between pairs of regions can be described using three different, but related, perspectives. These perspectives are anatomical or structural connectivity, functional connectivity and effective connectivity.

Functional connectivity estimate is typically based on the measurement of the temporal correlation in the activation between regions. This functional interaction can occur in task-dependent fMRI experiments, but also in resting-state fMRI experiments, as spontaneous low-frequency fluctuations of BOLD signal that indicate interaction between brain regions have been observed [2]. Resting-state fMRI images provide us with information about the default state of the brain, and allow us to evaluate functional connectivity and its alterations in brain disorders, such as ASD.

ASD is a neurological disorder whose prevalence has enormously increased in the last few years. Even though this is probably due to the improvements in the diagnostic methodology [6], this increase still remains concerning. It leads to look for new empirical approaches that contribute to a better understanding of the disorder, in order to develop new therapies that minimize the dysfunctions related to ASD. It remains unknown what the causes of the ASD are, although it is considered to be one of the neurodevelopmental disorders with a higher level of heritability [7]. Other risk factors that have been frequently observed in subjects with ASD are those associated with maternal gestational diabetes, and parents aged over 35 years [8].

There are two main behavioral patterns in ASD: the first is the deficiencies in communication and social interaction; the second is the restriction and repetitiveness of interests, activities and behaviors. Brain regions that appear to be altered in ASD are: (1) the default mode network (DMN), which is thought to be responsible of brain activity in resting-state [9]; (2) the mirror neuron system [10], which is intimately related to the “Theory of Mind” [11]; (3) the limbic system, which is involved in cognitive and emotional processing [12]; and (4) the motor system, with structures such as the basal ganglia and the cerebellum. Alterations in these structures may cause the imprecise and repetitive movements observed in ASD patients [13].

**Figure 1:**
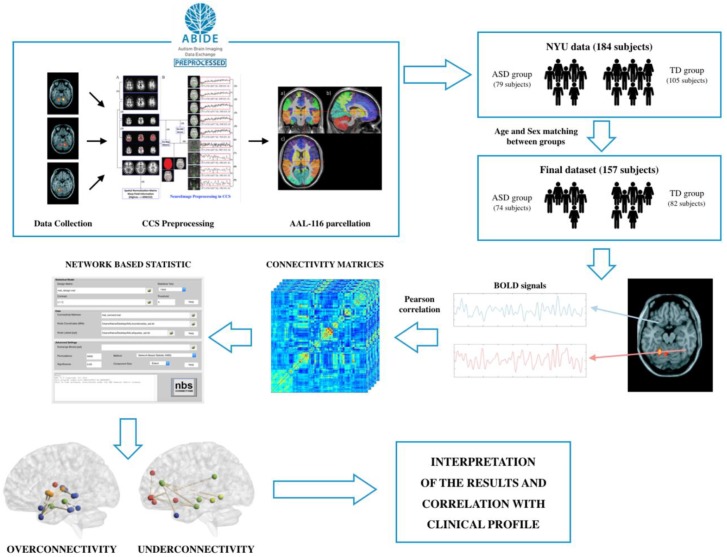
Framework of the study design. Firstly, fMRI images were obtained from ABIDE database (http://fcon_1000.projects.nitrc.org/indi/abide/). Then, ASD and TD groups were age and sex-matched, and functional brain networks were obtained using Pearson’s correlation coefficient between BOLD signals of every pair of brain regions. Using the toolbox “Network Based Statistic Toolbox” for Matlab, these networks were analyzed.

In this work, we have compared the connectivity patterns between ASD subjects and TD subjects, through the study of the spontaneous fluctuations of BOLD signals. When differences between these connectivity patterns have been found, we have attempted to provide a clinical meaning to these alterations in order to establish a relation between the obtained results and the clinical profile of the disorder. The framework of the study design is shown in Figure 1, whereas the data analysis procedures are detailed in Section 2.

## 2. Materials and Methods

### 2.1. Data Collection: Autism Brain Imaging Data Exchange

Functional MRI data were obtained from the Autism Brain Imaging Data Exchange (ABIDE) database. ABIDE is an initiative that has created an open bank of neuroimages to study ASD and contains images from 1112 subjects examined across different acquisition sites, with the intention of further contributing to the analysis and understanding of this disorder [14]. In this work, we used a set of images pertaining to 172 subjects of the New York University (NYU) dataset, of whom 75 are ASD subjects and 97 are TD subjects. Most of the subjects included in this study are children or adolescents and, in the case of the ASD group, they are high-functioning subjects. ABIDE database preprocesses the images according to several pipelines. In this work, the images analyzed were those preprocessed with the Connectome Computation System (CCS) pipeline [15].

The structural preprocessing stream starts with a noise reduction procedure, using a Non-Local Means filter. Then the skull is removed and the brain is segmented into white matter, gray matter and cerebrospinal fluid. Finally, the structural image is normalized to MNI (Montreal Neurological Institute) space, a standardized representation of human brain built from MRI images of 152 control subjects. This representation has been accepted as an international standard by the International Consortium of Brain Mapping (ICBM) with the name “ICBM152” [16].

The functional preprocessing pipeline consists of several steps. Firstly, the first four volumes of functional images are discarded, the spikes are removed and a 3D head motion correction is performed. Then, the global signal is extracted using a brain mask, the intensity is normalized and functional images are registered to the anatomical image. The brain-extracted anatomical image is then used to segment functional images into white matter, gray matter and cerebrospinal fluid. Afterwards, global signal regression and band-pass filtering between 0.01 and 0.1 Hz were applied. Finally, functional images are normalized to the MNI space. Further details as to the preprocessing pipeline can be found at http://preprocessed-connectomes-project.org/abide/Pipelines.html.

ABIDE database also provides several brain atlases that divide the brain into regions-of-interest (ROIs) according to anatomical and/or functional criteria. In this work, we used the Automated Anatomical Labeling (AAL) atlas, which divides the brain into 116 regions according to structural criteria [17]. This atlas includes regions of the four main lobes of the brain (frontal, parietal, temporal and occipital), as well as subcortical regions including the cingulate gyrus, the basal ganglia, and the cerebellum.

We further performed several analyses to ensure that there were not significant differences in “age” and “sex” between both groups. An initial analysis showed that there were not significant differences in variable “age” (*p*-value = 0.3065, two sample *t*-test), but these differences did appear in variable “sex” (*p*-value = 0.0313, *X*^2^-test). To correct these differences, 15 subjects of the TD group were removed from the dataset, obtaining a final dataset of 157 subjects: 75 ASD subjects and 82 TD subjects. With this final dataset, there were not significant inter-group differences in variable “age” (*p*-value = 0.3935) nor in variable “sex” (*p*-value = 0.9881). Table 1 lists the demographic information of subjects in both groups before and after the balance of variables “sex” and “age”.

Table 2 represents two indicators that reflect the performance of the ASD subjects in our dataset in different tests and scales used to evaluate ASD: on the one hand, the Autism Diagnostic Interview–Reviewed (ADI-R), and on the other hand, the Autism Diagnostic Observation Schedule (ADOS).

In Table 3 we have summarized the information regarding to both groups subjects’ intelligence quotient (IQ) level. We have performed a *t*-test to determine if there existed differences between them in the different fields in which IQ is evaluated. The results showed that there are no significant differences between both groups in Full-scale IQ Standard score nor in Performance IQ Standard Score, but there are differences in Verbal IQ Standard Score, what can be related to the difficulties that ASD subjects endure in the field of communication. IQ scores for both groups are normal in all fields.

### 2.2. Construction of Functional Brain Networks

**Figure 2:**
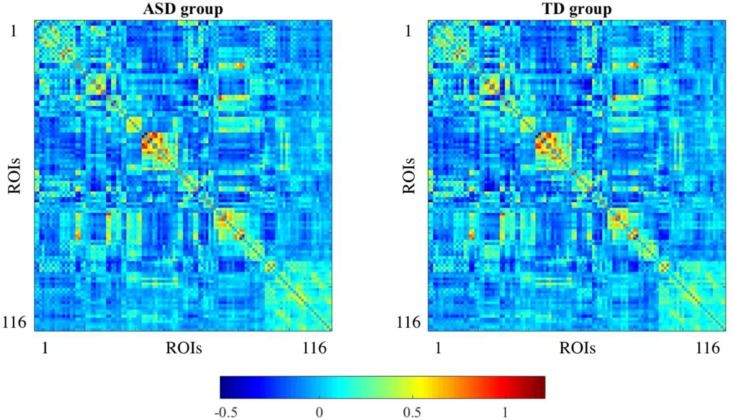
Mean connectivity matrices for ASD and TD groups. Connectivity is obtained using Pearson’s correlation coefficient normalized to Fisher’s *z*-values. Color scale represents the strength of functional interactions between pairs of ROIs.

We used MATLAB^®^ 2015a (The MathWorks, Inc., Natick, MA, USA) for network reconstruction and analysis. To evaluate functional connectivity alterations between ASD and TD subjects, we first extracted subject-specific connectivity networks, which are a set of 116 × 116 matrices that store the connectivity between each pair of nodes or brain regions, by computing the Pearson’s correlation coefficient of the BOLD signal between BOLD time courses. This connectivity measure can potentially take values in the interval [−1,1] After extracting the correlation coefficient for each pair of brain regions and for each one of the subjects, those values were normalized to Fisher’s *z*-values [18]. Figure 2 shows the mean connectivity matrix for each of the groups of the study (ASD and TD).

### 2.3. Network Analysis

To extract subnetworks or topological clusters of regions that are significantly differently connected between groups, we used the “Network Based Statistic Toolbox v1.2” (NBS toolbox) (The University of Melbourne, Melbourne, Australia) [19]. This toolbox has been specifically designed to test hypothesis in the connectome and search for differences either in inter-group connectivity or associated with an experimental effect. This methodology has been previously used to investigate the neurological basis of other neurological disorders, such as Borderline disorder [20]. In this work, we tested the hypotheses of both over connectivity (ASD > TD) and underconnectivity (ASD < TD) in ASD subjects [3]. First, the corresponding hypothesis is tested in every single connection using a two-sample *t*-test. The resulting test value *t* is compared to a pre-specified *t*-test threshold or primary threshold in every connection. The connections that exceed this threshold are those susceptible of showing significant differences in functional connectivity between ASD and TD subjects.

Afterwards, topological clusters are extracted. Finally, a *p*-value is computed for each cluster using permutation testing, and subsequently compared with a secondary threshold. If the obtained *p*-value for a cluster is lower than the secondary threshold, this cluster is considered as an altered network, in which the connectivity is significantly increased or decreased in ASD subjects, depending on whether one is testing the hypothesis of overconnectivity and underconnectivity, respectively. The secondary threshold, also referred as *p*-value threshold, was set to 0.025 (0.05/2), whereas the number of iterations to 5000.

After obtaining the significant networks, we made use of the Matlab toolbox “BrainNet Viewer” [21] to represent the altered network. Brain regions were grouped in the four main lobes of the brain, as well as in several structures that are susceptible to appearing in an altered form in ASD.

## 3. Results

### 3.1. Setting the Primary Threshold

In order to set the primary threshold, we tested different threshold values ranging from 1.5 to 4 in increments of 0.5 (Table 4). The other parameters of the method remained unaltered. Finally, a threshold value of 3.5 was set, as it provided us with an appropriate network size that contributed to the interpretation of the results.

### 3.2. Overconnectivity in ASD

**Figure 3:**
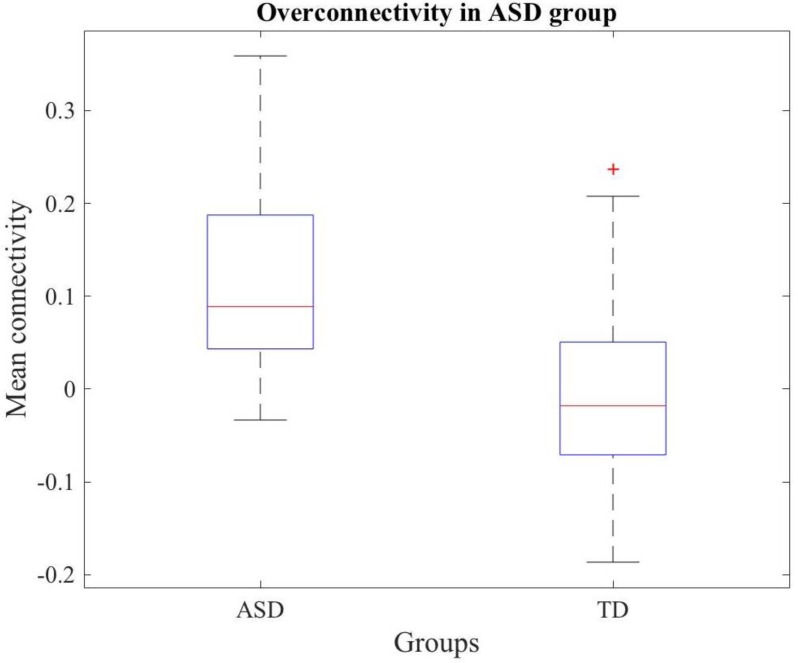
Box and Whiskers diagram representing the mean connectivity in ASD and TD groups for the different connections comprised in the network obtained under the overconnectivity hypothesis.

First, we tested the hypothesis of overconnectivity in ASD, and a network with significantly increased connectivity in the ASD group was observed. Figure 3 shows a Box and Whiskers diagram that represents the connectivity of the network for ASD and TD groups.

**Figure 4:**
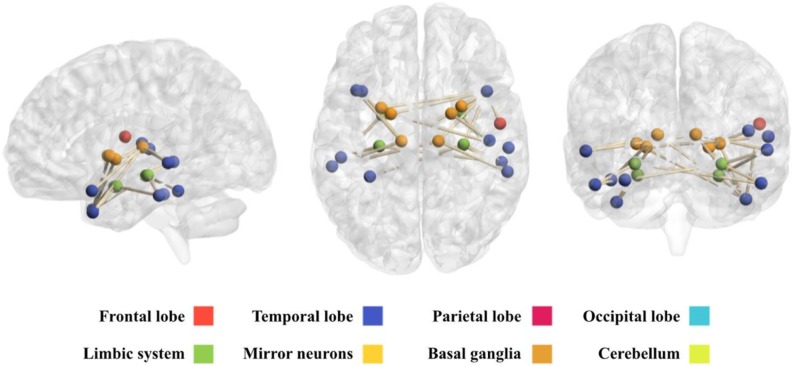
Representation of the network obtained under the overconnectivity hypothesis. From left to right: sagittal, axial and coronal views of the brain. Representation obtained with the BrainNet Viewer Toolbox [21].

This network comprises 21 brain regions that are connected through 24 edges (Table A1 in Appendix A). Figure 4 shows a view of this network, obtained with the BrainNet Viewer toolbox [21]. It can be observed that this network mainly includes regions pertaining to the temporal lobe, limbic system and basal ganglia.

### 3.3. Underconnectivity in ASD

**Figure 5:**
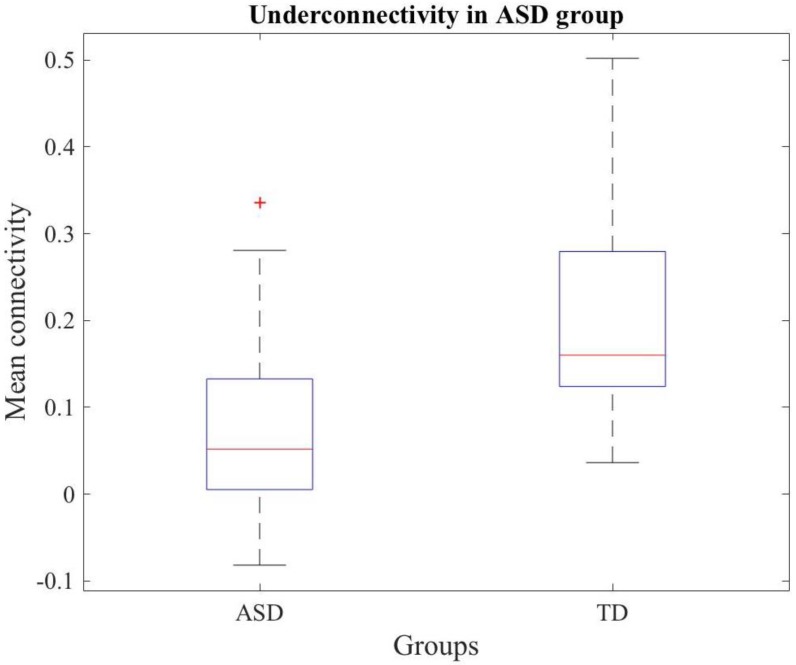
Box and Whiskers diagram representing the mean connectivity in ASD and TD groups for the different connections comprised in the network obtained under the underconnectivity hypothesis.

When testing the hypothesis of underconnectivity in ASD, a network that shows decreased connectivity in ASD subjects in comparison to TD subjects was observed. Figure 5 shows a Box and Whiskers diagram that represents the connectivity of the network for ASD and TD groups. 

**Figure 6:**
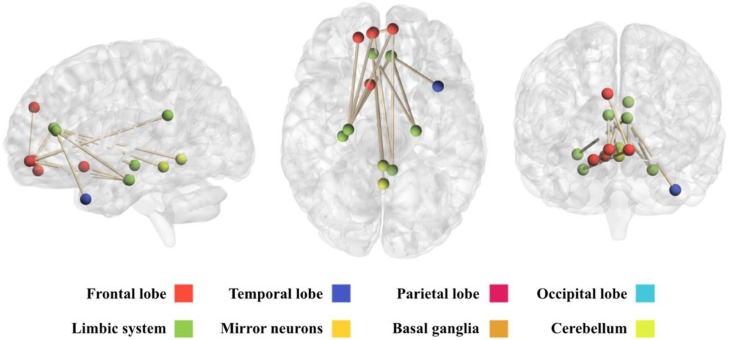
Representation of the network obtained under the underconnectivity hypothesis. From left to right: sagittal, axial and coronal views of the brain. Representation obtained with the BrainNet Viewer Toolbox [21].

This network comprises 14 brain regions that are connected among them through 14 edges (Table A2 in Appendix A). Figure 6 shows a view of this network, obtained with the BrainNet Viewer toolbox [21]. It can be observed that this network mainly involves regions pertaining to the limbic system and the frontal lobe.

## 4. Discussion

### 4.1. Different Methodologies of Network Science. NBS Limitations

In this study, we have used NBS to determine the differences in functional connectivity between ASD and TD subjects. This methodology focuses on the value of Pearson’s correlation coefficient between two brain regions to determine the regions that are over- or underconnected. It provides us with a network that comprises a group of regions that, as a whole, show an alteration in connectivity in ASD patients. 

Another approach to be used to determine network alterations in ASD subjects would be that of Graph theory. Graph theory offers a wide range of measures that characterize the topology of the reconstructed networks. As Bullmore and Sporns described in [5], some of the most used measures in brain networks analysis are node degree, degree distribution, clustering coefficients, path length, efficiency, centrality or modularity. Using this approach, they describe the brain network as a *small-world* network with a short path length, which is associated with a high efficiency of information transfer, and high clustering, which is associated to robustness to error. 

There are some limitations with our methodology. The first limitation of the NBS methodology is that it does not provide us with information of every individual connection, but gives us information about the behavior of the network as a whole [19]. Thus, we cannot interpret the relation between two particular brain regions in the network. However, NBS takes advantage of the fact that the different brain regions are interconnected, as many neurological disorders affect more than one region or edge. Moreover, the use of this methodology simplifies the interpretation of the results, as well as the establishment of relations with ASD clinical profile.

Another limitation of the NBS methodology is that it implies choosing a primary threshold that will inevitably condition the results. In this case, we have chosen a primary threshold of 3.5, as it provided us with smaller networks that were simpler to interpret and that showed the activities of some less-known brain regions in the networks. However, due to this choice, other brain regions that may also play an important role in ASD behavioral patterns, and that appear in bigger networks obtained with lower thresholds, do not appear when using a higher threshold of 3.5. 

This is the case, for instance, of the cerebellum. This brain region appears to be altered in networks obtained with a primary threshold ≤ 3. Networks obtained with this threshold show a high presence of cerebellum nodes (nodes 91–116 in AAL parcellation), whose alteration is considered to be responsible of the motor alterations that are characteristic of ASD patients [22].

In addition, our approach—network science from a ‘macro’ perspective—led us to choose a brain parcellation, which also conditions the results, as different brain parcellation schemes based on different criteria will not produce the same outputs, as they do not have the same number nor distribution of brain regions [23].

Finally, global signal regression (GSR) has been used for data preprocessing steps. The use of GSR is controversial, because it induces the appearance of negative correlations that can hardly be interpreted due to a lack of knowledge about mechanisms that operate these low-frequency BOLD signal fluctuations, and their relation with the associated neurological activity [24]. For this reason, the obtained results must be cautiously interpreted. 

### 4.2. Overconnectivity in ASD

An altered network was found under the overconnectivity hypothesis, including brain regions located in the temporal lobe, with increased connectivity with the limbic system and the basal ganglia. One of the most relevant regions of this network is the medial temporal gyrus. Even though the function of this region is not completely known, it is considered to be involved in processes such as distance evaluation, facial recognition or interpretation of the meaning of the words during reading [25]. It is noteworthy to highlight that this region does not show connectivity with the occipital lobe, which receives and processes visual stimuli, and with the frontal lobe, which processes the information in a rational way, comparing it to previous knowledge [26]. People with ASD possess considerable difficulties when they need to recognize faces and face expressions [27]. However, the presence of overconnectivity in the medial temporal gyrus is remarkable, as ASD subjects’ response to face identification is significantly slower than TD subjects’ response [28]. Other fMRI studies in ASD subjects have also established a relation between the activation of inferior and medial temporal gyrus, and the differences that these subjects experiment when processing faces [29]. 

On the other hand, the medial temporal gyrus is highly connected to basal ganglia and limbic system nodes. Those regions regulate key functions related to ASD behaviors: Basal ganglia have an important role in motor activity, and its alteration causes hyperkinetic and repetitive movements [13], whereas the limbic system regulates the instinctive response to stimuli, as well as the management of the emotions [12].

These difficulties in facial and facial expression recognition can be one of the main causes of the difficulties in social interaction that endure ASD subjects. The increase in connectivity between the temporal lobe and the regions from the limbic system and the basal ganglia can be explained as a difference in the brain circuitries that would be typical in TD subjects. These typical circuitries would involve the occipital and frontal lobes, regions that are more developed and rational than systems such as the basal ganglia and the limbic systems, which show a more primitive response [12].

Other regions that appear in the network and could have a clinical significance are the left and right temporal poles (regions 87 and 88 of the AAL116 atlas). Temporal poles are involved in the dotation of meaning to auditory stimuli, and their alteration may cause an inability to extract semantical meaning from them [30]. 

Moreover, the network shows a strong presence of nodes pertaining to basal ganglia, such as the thalamus, putamen or pallidum. Basal ganglia are thought to be involved in motor system abnormalities that are observed in ASD children [31], such as the difficulties in planning and executing precise movements, gait abnormalities, spasmodic or involuntary movements, or repetitive and stereotyped movements, such as head banging, hand flapping or body rocking. Other studies have found both structural and metabolic alterations in basal ganglia, such as an increase in the volume of the caudate nucleus [32] or alterations in the metabolism of glucose in posterior putamen [33]. 

The global analysis of the network suggests that the functional alterations of the temporal lobe may cause the social interaction difficulties that affect ASD subjects. These results support the thesis of Boddaert et al. [34], who affirmed that the disruption of connections in the temporal lobe are one of the main causes of the socio-emotional dysfunctions of patients with ASD. The alteration of the traditional paths of information exchange in the temporal lobe can lead to the search of alterative paths, which makes ASD subjects manage differently the processing of visual stimuli, such as facial recognition, and auditory stimuli, such as the dotation of semantical meaning to the inputs that registers the auditory nerve. In addition, the alteration of connectivity in the basal ganglia can be related with the motor alterations that are observed in ASD patients, and that are one of the most identifiable characteristics of their behavioral pattern. 

### 4.3. Underconnectivity in ASD

It can be observed that the network obtained under the underconnectivity hypothesis mainly involves regions pertaining to the limbic system and the frontal lobe. Among the different frontal lobe regions that appear in the network, the presence of the orbitofrontal cortex (regions 25 and 26 of AAL116 atlas) can be highlighted. This brain area is supposed to be implicated in decision-making processes, especially in those related to reward and punishment conditions [35], which makes them strongly associated with an emotional response. The orbitofrontal cortex is therefore an area that plays a key role in adaptive learning. Hence, the disruption of the connectivity in the orbitofrontal cortex may cause the alteration of cognitive, emotional and behavioral processes [36]. 

These regions in the frontal lobe are strongly connected with the limbic system. The limbic system controls the instinctive emotional response in front of stimuli such as fear or pleasure [12]. It also develops an important role in adaptive learning through operant conditioning [37], which associates an action to a response that can cause in the subject either positive (e.g., pleasure) or negative emotions (e.g., fear). Neurotypical subjects associate their previous experiences with the feelings that these experiences have caused on them. 

However, the disruption of the connectivity between the orbitofrontal cortex and the limbic system suggests that the emotions that experiment ASD subjects, which may be firstly processed by the limbic system, are not able to continue to the frontal lobe, where they would be compared with previous knowledge and then rationalized, performing a higher-level cognitive processing. This is concordant with the difficulties in the management of cognitive and affective processes that face ASD subjects, according to the description of the disorder provided in the DSM-V [38]. These difficulties are related with what is known as “Theory of Mind”. Olivito et al. [39] suggested that the disconnection of the regions that house the Theory of Mind may be responsible for the social impairments that characterize ASD patients. This theory states that humans, through the mirror neuron system, that is mainly located in the frontal lobe, as well as other regions in parietal lobe or cerebellum, are able to identify, understand and predict other people’s moods, emotions and actions. ASD patients are thought to be incapable of identifying this moods and emotions, and therefore, are not able to adopt other person’s point of view. This is what professor Simon Baron-Cohen described in 1985 as mind-blindness [11]. 

Furthermore, other fMRI studies that support the underconnectivity hypothesis in ASD patients consider that both the frontal lobe and the limbic system have an implication in the brain dysfunctions that ASD patients experience [33,34]. 

## 5. Conclusions

We used the network-based statistics approach to determine brain alterations in the context of ASD. The obtained results show alterations in brain regions involved in the management of affective and cognitive processes, and in motor control. These alterations are in accordance with ASD clinical profile. 

The obtained results suggest that the functional connectome of ASD subjects exhibits a combination of brain overconnectivity and underconnectivity hypothesis. However, these results should be interpreted cautiously, due to the controversy associated with the application of the global signal regression step in the preprocessing pipeline. In this work, negative correlations have been interpreted as a lower interaction between brain regions. 

The analysis of the differences in connectivity between brain regions permits to deepen in the establishment of the neurological bases of ASD, which can help improve its understanding and the development of specific therapies. Moreover, the methodology applied in this study shows great potential, and can be applied to the study of other neurological disorders, such as alcoholism and Alzheimer’s disease. 

## Figures and Tables

**Table 1:** Demographic information about subjects in ASD and TD groups.

Demographic Value	Original Dataset (172 Subjects)		Final Dataset (157 Subjects)	
	ASD	TD	ASD	TD
No. of subjects	75	97	75	82
Age (mean years ± std. deviation)	14.54 ± 6.95	15.56 ± 6.17	14.54 ± 6.95	15.40 ± 5.75
Age *p*-value (two sample *t*-test)	0.3065		0.3935	
Sex (no. of males and females)	♂ 65	♂ 71	♂ 65	♂ 71
	♀ 10	♀ 26	♀ 10	♀ 11
Sex *p*-value (*X*^2^)	0.0313		0.9881	

**Table 2:** Indicators of performance of ASD subjects in different fields of behavior that allow clinicians to better evaluate the disorder.

Performance Indicator	ADI-R	ADOS
Social Interaction Subscore	18.9242 ± 5.6113	7.7733 ± 3.0205
Communication Subscore	15.8030 ± 4.5513	3.5600 ± 1.5875
Restricted and Repetitive Behaviors Subscore	5.6061 ± 2.6711	2.3867 ± 1.5500

**Table 3:** Different IQ Scores for ASD and TD subjects. A two-sample *t*-test has been performed in order to determine the existence of significant differences in any evaluated fields. Results show that there exist differences in Verbal IQ Score between both groups.

IQ Score	ASD	TD	*p*-Value (Two-Sample *t*-Test)
Full-Scale IQ Standard Score	107.8267 ± 16.6987	112.4533 ± 12.1781	0.0544
Verbal IQ Standard Score	105.3600 ± 16.0621	112.3200 ± 11.3235	0.0026
Performance IQ Standard Score	109.0933 ± 17.6085	109.7333 ± 13.8049	0.8047

**Table 4:** Number of nodes and links of the subnetwork obtained under the hypothesis of overconnectivity (OC) in ASD patients and underconnectivity (UC) in ASD patients, under different primary thresholds in NBS test. In all the significant cases, the *p*-value was lower than 0.01.

Primary Threshold	Hypothesis	No. of Nodes	No. of Links
*t* = 1.5	OC	116	923
	UC	116	836
*t* = 2	OC	112	480
	UC	113	429
*t* = 2.5	OC	97	228
	UC	89	201
*t* = 3	OC	57	87
	UC	48	71
*t* = 3.5	OC	21	24
	UC	14	14
*t* = 4	OC	12	11
	UC	*No significant result*	

## Appendix A

**Table A1:** List of the 24 links comprised in the overconnectivity network.

Node A			Node B		
No.	Tag		No.	Tag	
38	•	‘Hippocampus_R’	55	•	‘Fusiform_L’
76	•	‘Pallidum_R’	80	•	‘Heschl_R’
75	•	‘Pallidum_L’	82	•	‘Temporal_Sup_R’
77	•	‘Thalamus_L’	83	•	‘Temporal_Pole_Sup_L’
77	•	‘Thalamus_L’	85	•	‘Temporal_Mid_L’
78	•	‘Thalamus_R’	85	•	‘Temporal_Mid_L’
38	•	‘Hippocampus_R’	86	•	‘Temporal_Mid_R’
42	•	‘Amygdala_R’	86	•	‘Temporal_Mid_R’
78	•	‘Thalamus_R’	86	•	‘Temporal_Mid_R’
41	•	‘Amygdala_L’	87	•	‘Temporal_Pole_Mid_L’
77	•	‘Thalamus_L’	87	•	‘Temporal_Pole_Mid_L’
18	•	‘Rolandic_Oper_R’	88	•	‘Temporal_Pole_Mid_R’
38	•	‘Hippocampus_R’	88	•	‘Temporal_Pole_Mid_R’
42	•	‘Amygdala_R’	88	•	‘Temporal_Pole_Mid_R’
73	•	‘Putamen_L’	88	•	‘Temporal_Pole_Mid_R’
74	•	‘Putamen_R’	88	•	‘Temporal_Pole_Mid_R’
75	•	‘Pallidum_L’	88	•	‘Temporal_Pole_Mid_R’
76	•	‘Pallidum_R’	88	•	‘Temporal_Pole_Mid_R’
77	•	‘Thalamus_L’	88	•	‘Temporal_Pole_Mid_R’
78	•	‘Thalamus_R’	88	•	‘Temporal_Pole_Mid_R’
37	•	‘Hippocampus_L’	89	•	‘Temporal_Inf_L’
41	•	‘Amygdala_L’	89	•	‘Temporal_Inf_L’
37	•	‘Hippocampus_L’	90	•	‘Temporal_Inf_R’
38	•	‘Hippocampus_R’	90	•	‘Temporal_Inf_R’

**Table A2:** List of the 14 links comprised in the underconnectivity network.

Node A			Node B		
No.	Tag		No.	Tag	
24	•	‘Frontal_Sup_Medial_R’	25	•	‘Frontal_Med_Orb_L’
26	•	‘Frontal_Med_Orb_R’	32	•	‘Cingulum_Ant_R’
25	•	‘Frontal_Med_Orb_L’	35	•	‘Cingulum_Post_L’
26	•	‘Frontal_Med_Orb_R’	35	•	‘Cingulum_Post_L’
31	•	‘Cingulum_Ant_L’	38	•	‘Hippocampus_R’
31	•	‘Cingulum_Ant_L’	39	•	‘ParaHippocampal_L’
32	•	‘Cingulum_Ant_R’	39	•	‘ParaHippocampal_L’
6	•	‘Frontal_Sup_Orb_R’	40	•	‘ParaHippocampal_R’
22	•	‘Olfactory_R’	40	•	‘ParaHippocampal_R’
25	•	‘Frontal_Med_Orb_L’	40	•	‘ParaHippocampal_R’
26	•	‘Frontal_Med_Orb_R’	40	•	‘ParaHippocampal_R’
31	•	‘Cingulum_Ant_L’	87	•	‘Temporal_Pole_Mid_L’
32	•	‘Cingulum_Ant_R’	110	•	‘Vermis_3’
32	•	‘Cingulum_Ant_R’	111	•	‘Vermis_4_5’
